# Synergistic Enhancements in Maize Yield and Water Productivity: Dense Planting with Regulated Deficit Irrigation Versus Conventional Practice

**DOI:** 10.3390/plants15121880

**Published:** 2026-06-17

**Authors:** Feng Wang, Haofeng Meng, Jun Xue, Bo Ming, Guoqiang Zhang, Keru Wang, Ruizhi Xie, Shaokun Li

**Affiliations:** 1 State Key Laboratory of Aridland Crop Science, Seed Industry Research Institute, College of Agronomy, Gansu Agricultural University, Lanzhou 730070, China; wangfen@gsau.edu.cn; 2 Key Laboratory of Crop Physiology and Ecology, Ministry of Agriculture and Rural Affairs, Institute of Crop Sciences, Chinese Academy of Agricultural Sciences, Beijing 100081, China; xuejun@caas.cn (J.X.); mingbo@caas.cn (B.M.); zhangguoqiang@caas.cn (G.Z.); wangkeru@caas.cn (K.W.); xieruizhi@caas.cn (R.X.)

**Keywords:** dense planting, regulated deficit irrigation, water productivity, synergistic effect, soil water threshold, arid agroecosystems

## Abstract

Enhancing both grain yield and water productivity (WP) under water scarcity is a critical challenge for sustainable maize production. Conventional farmer practices—characterized by low planting density (D_1_: 9.0 × 10^4^ plants·ha^−1^) and excessive irrigation (I_5_)—often limit the potential for both. To advance the high-density production system enabled by precision stage-specific regulation (HD-PSR), we conducted a multi-region field experiment across arid to sub-humid climates in Xinjiang, China. Treatments included region-specific full irrigation (I_5_) and four deficit levels (I_1_–I_4_, each reduced by 90 mm relative to I_5_). Compared with the conventional model (D_1_ + I_5_), the HD-PSR-based optimized model (D_2_, 12.0 × 10^4^ plants·ha^−1^ plus regulated deficit irrigation, I_opt_) synergistically increased grain yield by 18.8–25.0% and WP by 8.7–20.0% across sites, while saving 11.1–50.0% of irrigation water. This synergy was driven by improved canopy coverage and sustained dry matter accumulation under precision-regulated irrigation that maintained soil water storage at ~70% of field capacity, thereby reducing non-productive water losses (soil evaporation in arid/semi-arid areas and excessive transpiration in the semi-humid area). Our study provides a quantitative irrigation module for the HD-PSR and identifies a translatable soil water threshold (~70% FC) for sustainable intensification of maize in water-limited regions.

## 1. Introduction

Achieving food security under severe water scarcity is a pressing global challenge, exemplified by China’s need to feed nearly 20% of the world’s population with limited water and arable land. In Northern China, where most farmland depends on irrigation amid growing water stress and extreme droughts [1,2], improving crop water productivity (WP) through efficient water use has become a national priority.

Maize is a key crop for global food and feed production and is highly sensitive to water scarcity. High-density planting is widely recognized for increasing yield [3,4,5] and has been successfully adopted in Northwest China, often combined with drip irrigation under plastic mulch [6,7]. However, the combined effect of dense planting and water management remains poorly quantified. In practice, high-density systems are frequently paired with traditional over-irrigation, which elevates evapotranspiration (ET) without proportional yield gain, thereby lowering WP [8,9,10]. Most previous studies have examined density and water management in isolation or within single environments [11,12,13], leaving critical gaps—the synergistic effect, i.e., a combined treatment producing greater improvements in yield and WP than the sum of individual effects, has not been systematically quantified across contrasting climates, and a translatable physiological indicator (e.g., a soil water threshold) that could universally support high yield and WP is still lacking.

Enhancing water productivity (WP) fundamentally depends on shifting evapotranspiration (ET) partitioning towards productive plant transpiration (T) and away from non-productive soil evaporation (E) [14]. Film mulching is known to favorably shift this balance toward T [15,16,17,18,19]. However, under high planting density, the physiological mechanisms by which precision irrigation actively modulates T/E partitioning—and how this modulation coordinates canopy development, dry matter accumulation, and microclimate—remain inadequately understood across diverse agroecosystems.

This study is positioned within the emerging high-density production system enabled by a precision stage-specific regulation (HD-PSR) framework. The HD-PSR approach integrates increased plant density with growth stage-specific irrigation regulation to maximize both yield and WP. We hypothesize that replacing the conventional practice (low density + over-irrigation) with an optimized practice (high density + regulated deficit irrigation) will (1) maintain high yield through stable canopy photosynthesis and dry matter partitioning, and (2) synergistically enhance WP by optimizing soil water conditions to reduce non-productive water loss. The specific objectives were to (i) quantify yield and WP differences between conventional and optimized production modes across an aridity gradient; (ii) elucidate the physiological mechanisms (e.g., dry matter accumulation, canopy cover, soil water, E/T partitioning) underlying any synergistic effect; and (iii) identify a generalizable soil water threshold that sustains both high yield and WP, thereby providing a quantitative irrigation module for the scalable HD-PSR framework.

## 2. Results

### 2.1. Grain Yield

**Figure 1:**
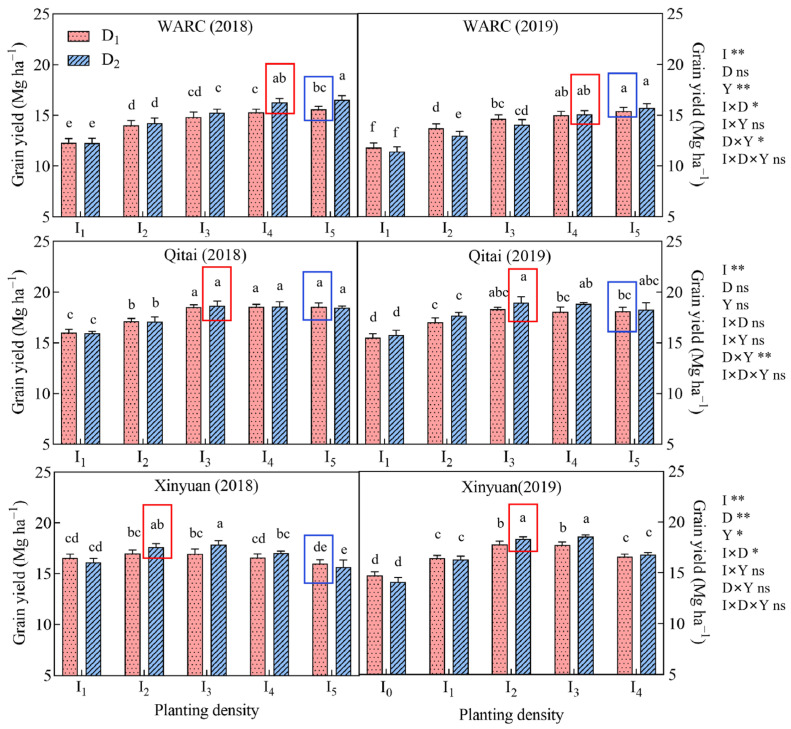
Grain yield of maize under different irrigation amounts at WARC, Qitai, and Xinyuan. Different letters within a column stand for significant differences at *p* < 0.05: I, irrigation volume; D, planting density; Y, year. * mean significance at *p* < 0.05; ** mean significance at *p* < 0.01; ns, no significance. Blue boxes represent the values of the farmers’ planting patterns, and red boxes represent the values of the optimization patterns.

Under the conventional farmer pattern (D_1_ + I_5_), grain yields varied significantly across sites (*p* < 0.05), with the lowest at the arid site (WARC) and the highest at the semi-arid site (Qitai) (Figure 1). Increasing planting density to D_2_ with reduced irrigation (I_4_ at WARC, I_3_ at Qitai, I_2_ at Xinyuan) caused no significant yield reduction at any site (*p* > 0.05); under the optimized pattern (D_2_ + I_opt_), yield increased by 1.3% at WARC and 1.6% at Qitai (both non-significant) but by 12.5% at Xinyuan (*p* < 0.05) (Figure 1). Significant irrigation × density interactions at WARC and Xinyuan (*p* < 0.05) indicate a non-additive effect, where dense planting raises water demand, and precision deficit irrigation meets it without excess, avoiding yield loss. The larger significant increase at the semi-humid site (Xinyuan) likely reflects that excessive conventional irrigation (I_5_) promoted redundant vegetative growth and a reduced harvest index, while reducing irrigation to I_2_ redirected assimilates to grain filling; conversely, at the arid and semi-arid sites, high evaporative demand and limited rainfall constrained the yield response. Significant density × year interactions at WARC and Qitai (*p* < 0.05) further suggest that inter-annual climate variability modulates high-density system performance, underscoring the need for climate-adaptive irrigation scheduling. Because the irrigation × density interaction was significant at WARC and Xinyuan, multiple comparisons (Duncan’s test) identified the optimal treatment combination at each site as D_2_ + I_4_ at WARC (15.7 Mg ha^−1^, not significantly different from D_1_ + I_5_), D_2_ + I_3_ at Qitai (18.6 Mg ha^−1^, ns), and D_2_ + I_2_ at Xinyuan (18.0 Mg ha^−1^, *p* < 0.05 vs. D_1_ + I_5_), which represent the optimized model (D_2_ + I_opt_) used in subsequent analyses (Figure 1).

**Figure 2:**
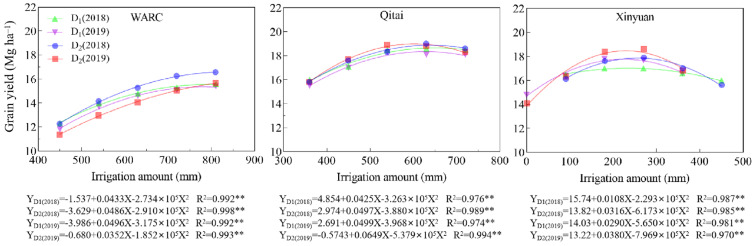
Quadratic functions fitted to the relationships between grain yields (y) and irrigation amounts (x) at Changji, Qitai, and Xinyuan. ** mean significance at *p* < 0.01.

The relationship between grain yield and irrigation amount followed a quadratic function (R^2^ > 0.970, *p* < 0.05; Figure 2). At Qitai and Xinyuan, the fitted curves reached clear maxima within the tested irrigation range, giving optimal irrigation amounts for maximum yields of 535.0–554.4 mm (D_1_) and 603.0–640.2 mm (D_2_) at Qitai, and 224.7–236.2 mm (D_1_) and 229.0–255.6 mm (D_2_) at Xinyuan. At WARC, while the quadratic model estimated optimal irrigation amounts of 780.0–792.2 mm (D_1_) and 834.4–950.9 mm (D_2_), the yield under D_2_ continued to rise across the tested range (up to I_5_ = 810 mm) without reaching a plateau; therefore, the true optimum for D_2_ at WARC likely exceeds 810 mm. Increasing planting density by 3 × 10^4^ plants·ha^−1^ raised the optimal irrigation requirement by 106 mm (WARC, estimated), 77 mm (Qitai), and 17 mm (Xinyuan) (Figure 2).

### 2.2. Water Productivity

**Figure 3:**
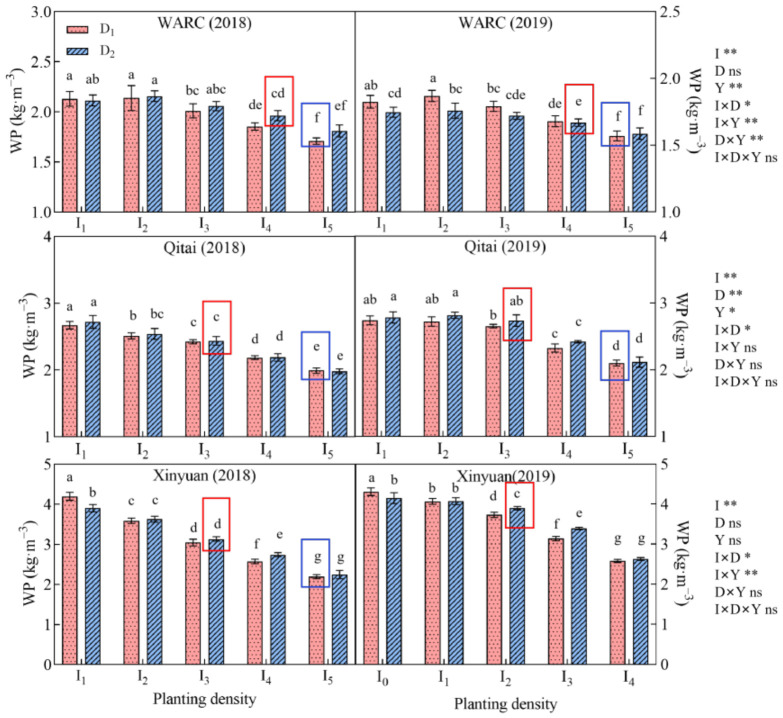
Water productivity (WP) of maize under different irrigation amounts at WARC, Qitai, and Xinyuan. Different letters within a column stand for significant differences at *p* < 0.05: I, irrigation volume; D, planting density; Y, year. * mean significance at *p* < 0.05; ** mean significance at *p* < 0.01; ns, no significance. Blue boxes represent the values of the farmers’ planting patterns, and red boxes represent the values of the best patterns.

At the arid site (WARC), WP under the conventional farmer pattern (D_1_ + I_5_) was significantly lower than at the semi-arid and semi-humid sites (*p* < 0.05, Figure 3). By contrast, the optimized pattern (D_2_ + I_opt_: I_4_ at WARC, I_3_ at Qitai, I_2_ at Xinyuan) produced significantly higher WP across all locations relative to D_1_ + I_5_ (*p* < 0.05), with gains of 10.8% (WARC), 26.4% (Qitai), and 71.5% (Xinyuan) (Figure 3). A significant irrigation × density interaction for WP was evident at all sites (*p* < 0.05), demonstrating a non-additive synergy, where higher density elevates crop water demand, while regulated deficit irrigation meets this demand without excess and redirects water use from evaporation toward transpiration, thereby improving WP. The particularly large increase at Xinyuan (71.5%) reflects the greatest reduction of over-irrigation under conventional practice at this semi-humid site (Figure 3). Given the significant interaction, Duncan’s multiple comparisons were used to select the optimal WP treatment combination, which coincided with D_2_ + I_opt_ already identified for yield (WARC: D_2_ + I_4_; Qitai: D_2_ + I_3_; Xinyuan: D_2_ + I_2_). Thus, substituting dense planting plus optimized deficit irrigation for conventional practice offers an effective route to enhance water productivity in water-limited regions.

### 2.3. Dry Matter Accumulation

**Figure 4:**
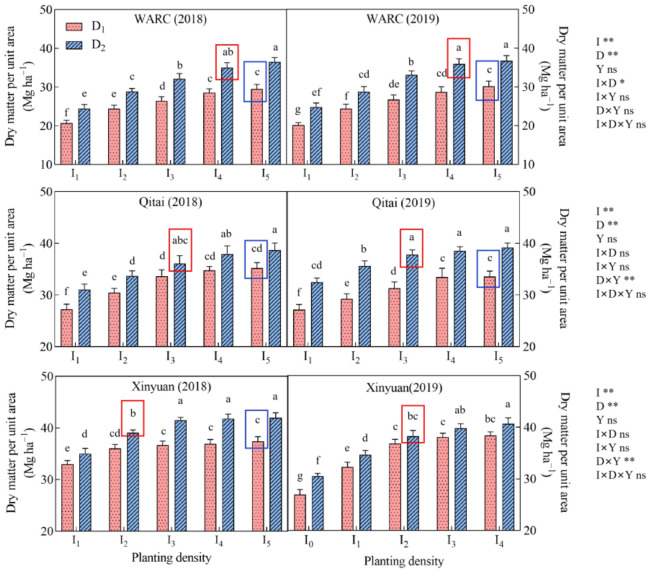
Maize dry matter per unit area at mature stage under different treatments at WARC, Qitai, and Xinyuan. Different letters within a column stand for significant differences at *p* < 0.05: I, irrigation volume; D, planting density; Y, year. * mean significance at *p* < 0.05; ** mean significance at *p* < 0.01; ns, no significance. Blue boxes represent the values of the farmers’ planting patterns, and red boxes represent the values of the optimization patterns.

Under the conventional farmer pattern (D_1_ + I_5_), dry matter accumulation per unit area did not rank highest among treatments at any site, indicating that excessive irrigation failed to maximize biomass (Figure 4). Increasing planting density to D_2_ significantly enhanced dry matter, while reducing irrigation had the opposite effect. Nevertheless, the optimized model (D_2_ + I_opt_, i.e., I_4_ at WARC, I_3_ at Qitai, and I_2_ at Xinyuan) achieved the highest dry matter per unit area across all sites (Figure 4). Compared with D_1_ + I_5_, D_2_ + I_opt_ increased dry matter by 18.8% at WARC, 7.5% at Qitai, and 11.0% at Xinyuan, with the overall order of Xinyuan > Qitai > WARC (Figure 4). The relatively modest increase at the semi-arid site (Qitai, 7.5%) likely reflects water limitation constraining the synergistic response, whereas the larger increases at WARC (18.8%) and Xinyuan (11.0%) suggest that eliminating over-irrigation (arid site) or excessive vegetative growth (semi-humid site) allowed the density effect to be fully realized. Thus, under the optimized irrigation threshold, increased planting density effectively enhanced community-level dry matter productivity, providing a fundamental physiological basis for achieving higher yield (Figure 4).

**Figure 5:**
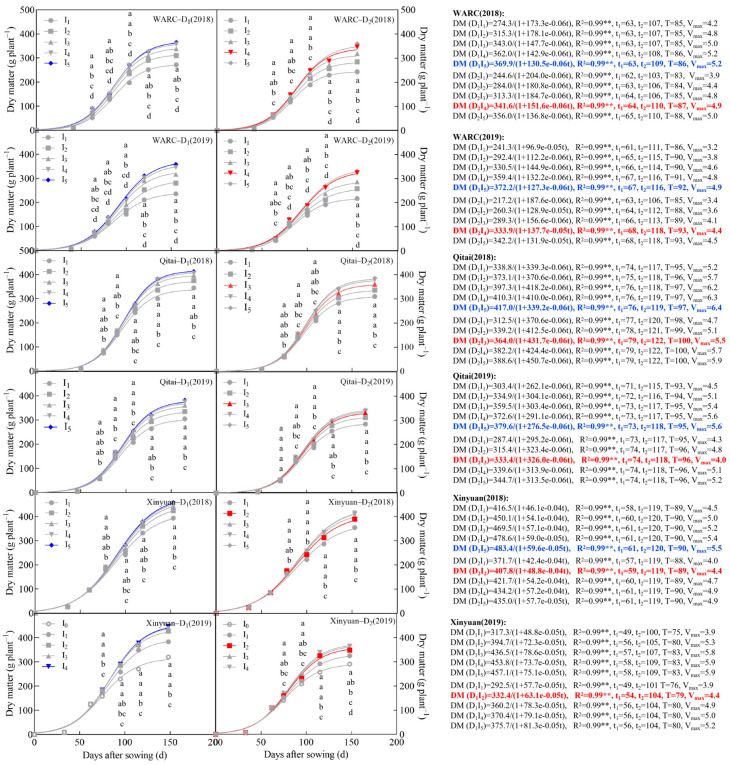
Maize dry matter simulated by a logistic formula under different treatments at WARC, Qitai, and Xinyuan. *t*_1_ and *t*_2_ are the initiation and termination times of rapid dry matter accumulation (d); *T* is the duration period of rapid dry matter accumulation (d); V_max_ is the maximum rate of rapid dry matter accumulation (g plant^−1^ d^−1^). Different lowercase letters indicate the level of significant difference (*p* < 0.05) among treatments at the same growth stage. ** mean significance at *p* < 0.01. Blue curves represent the values of the farmers’ planting patterns, and red curves represent the values of the optimization patterns.

The logistic formula successfully described the dry matter accumulation dynamics under different treatments (all fits with *R*^2^ > 0.99) (Figure 5). Key growth parameters revealed differences between the two production modes and across regions (Figure 5). Overall, the maximum accumulation rate per plant (Vmax) under the optimized model (D_2_ + I_opt_) was slightly lower than that under the farmer model (D_1_ + I_5_) at all sites; for example, decreasing from 5.2 to 4.8 g plant^−1^ d^−1^ at WARC, from 6.4 to 5.4 g plant^−1^ d^−1^ at Qitai, and significantly from 6.5 to 4.4 g plant^−1^ d^−1^ at Xinyuan. This reflects the inter-plant competition for resources under high-density conditions. However, the duration of the rapid accumulation phase (T) between its start (t_1_) and end (t_2_) times showed no significant differences between the two models. For instance, at WARC, the rapid accumulation phase lasted approximately 95–97 days under both models; at Qitai, the t_1_ values were 76 (D_1_ + I_5_) and 79 (D_2_ + I_opt_) days after sowing, and t_2_ values were 119 and 122 days, respectively (Figure 5). This indicates that optimized irrigation management, while saving water, ensured that the critical phenological window for rapid dry matter accumulation was not substantially shortened, providing a temporal foundation for the stable formation of final biomass. In summary, although the maximum growth rate per plant was reduced under the optimized model, it ultimately achieved a significant increase in total dry matter per unit area by maintaining a sufficient duration of the rapid accumulation phase and combining it with a higher plant population density (D_2_). This explains, from the perspective of growth dynamics, the physiological basis for yield stability under the synergistic “dense planting water saving” model.

### 2.4. Canopy Coverage (CC)

**Figure 6:**
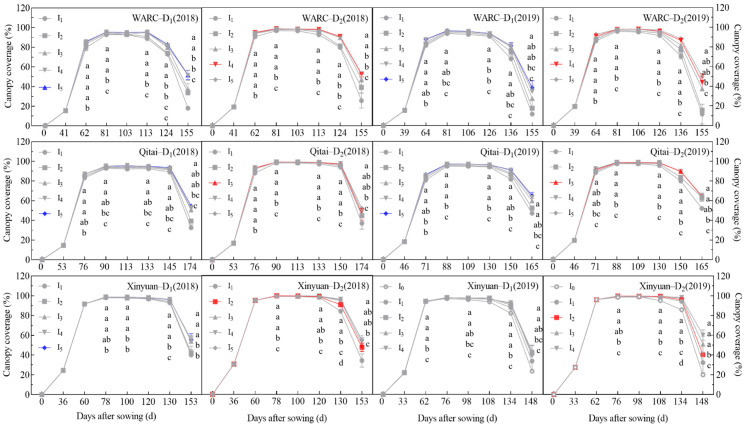
Canopy coverage of maize under different treatments at WARC, Qitai, and Xinyuan. Different lowercase letters indicate the level of significant difference (*p* < 0.05) among treatments at the same growth stage. Blue curves represent the values of the farmers’ planting patterns, and red curves represent the values of the optimization patterns.

**Figure 7:**
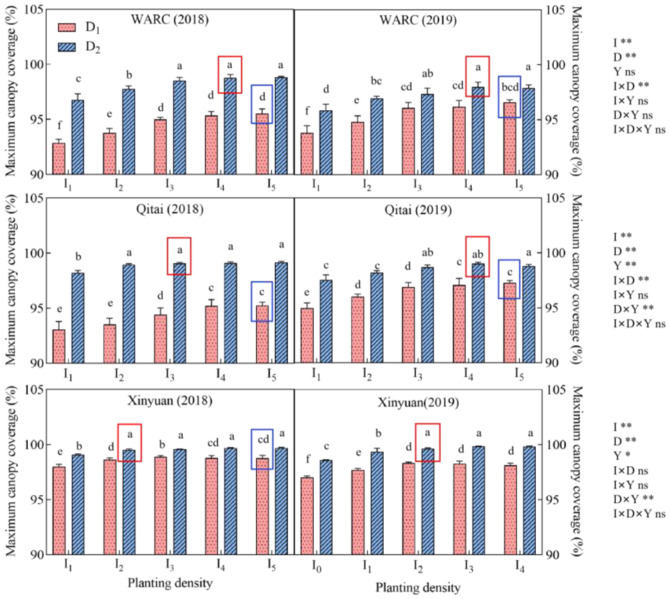
Maximum canopy coverage (CC_max_) at silking stage under different treatments at WARC, Qitai, and Xinyuan. Different letters within a column stand for significant differences at *p* < 0.05: I, irrigation volume; D, planting density; Y, year. * mean significance at *p* < 0.05; ** mean significance at *p* < 0.01; ns, no significance. Blue boxes represent the values of the farmers’ planting patterns, and red boxes represent the values of the optimization patterns.

Canopy coverage (CC) dynamics were significantly influenced by both irrigation amount and planting density (*p* < 0.05) (Figure 6). Across all sites, the combination of high planting density and full irrigation (D_2_ + I_5_) achieved the highest maximum CC at silking (>98.5%; Figure 7). Under the conventional farmer pattern (D_1_ + I_5_), maximum CC was significantly lower at the arid and semi-arid sites (WARC and Qitai) compared with the semi-humid site (Xinyuan) (*p* < 0.05). Compared with D_1_ + I_5_, the optimized model (D_2_ + I_opt_) significantly increased maximum CC at all three sites, with relative improvements of 1.7% at WARC, 2.7% at Qitai, and 0.7% at Xinyuan (*p* < 0.05).

### 2.5. Crop Evapotranspiration, Soil Evaporation and Maize Transpiration

**Figure 8:**
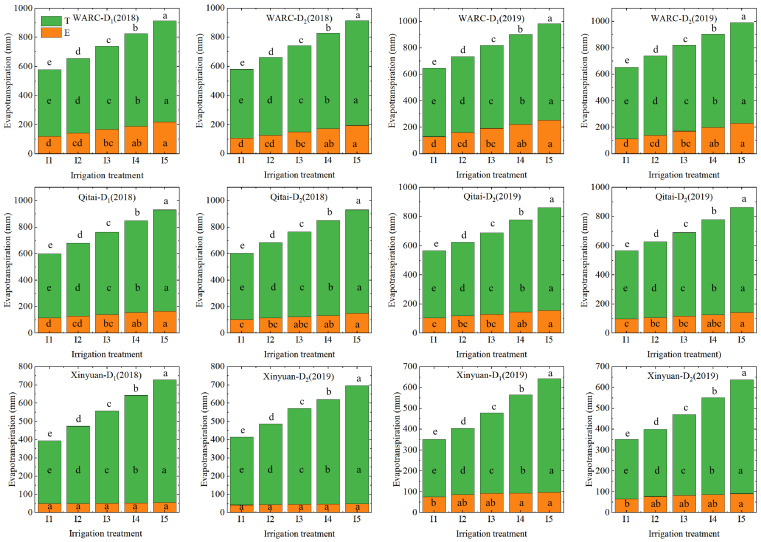
Crop evapotranspiration, transpiration (T) and soil evaporation (E) under different treatments at WARC, Qitai, and Xinyuan. Different letters within a column stand for significant differences at *p* < 0.05.

Across all sites, the conventional farmer pattern (D_1_ + I_5_) resulted in the highest evapotranspiration (ET), soil evaporation (E), and plant transpiration (T) compared with other treatments (*p* < 0.05; Figure 8). Under the optimized model (D_2_ + I_opt_), ET, E, and T were all significantly reduced relative to D_1_ + I_5_ (*p* < 0.05). The relative reductions were ET decreased by 8.7% at WARC, 23.0% at Qitai, and 30.2% at Xinyuan; E decreased by 21.0%, 26.4%, and 11.4%; and T decreased by 4.6%, 17.2%, and 31.5% (Figure 8). Notably, the proportional reduction in E was consistently smaller than that in T across all sites, leading to a lower E/T ratio under the optimized model. The results of the interaction analysis were as follows: At WARC, the interactions of year × planting density and year × irrigation had a significant effect on T and E. At Qitai, the interaction of year × irrigation significantly affected ET and T, while the interaction of planting density × irrigation significantly influenced ET and T. At Xinyuan, the interactions of year × planting density, year × irrigation, and year × planting density × irrigation all showed significant effects on T (Table 1).

### 2.6. Soil Water Storage and Layered Water Content

**Figure 9:**
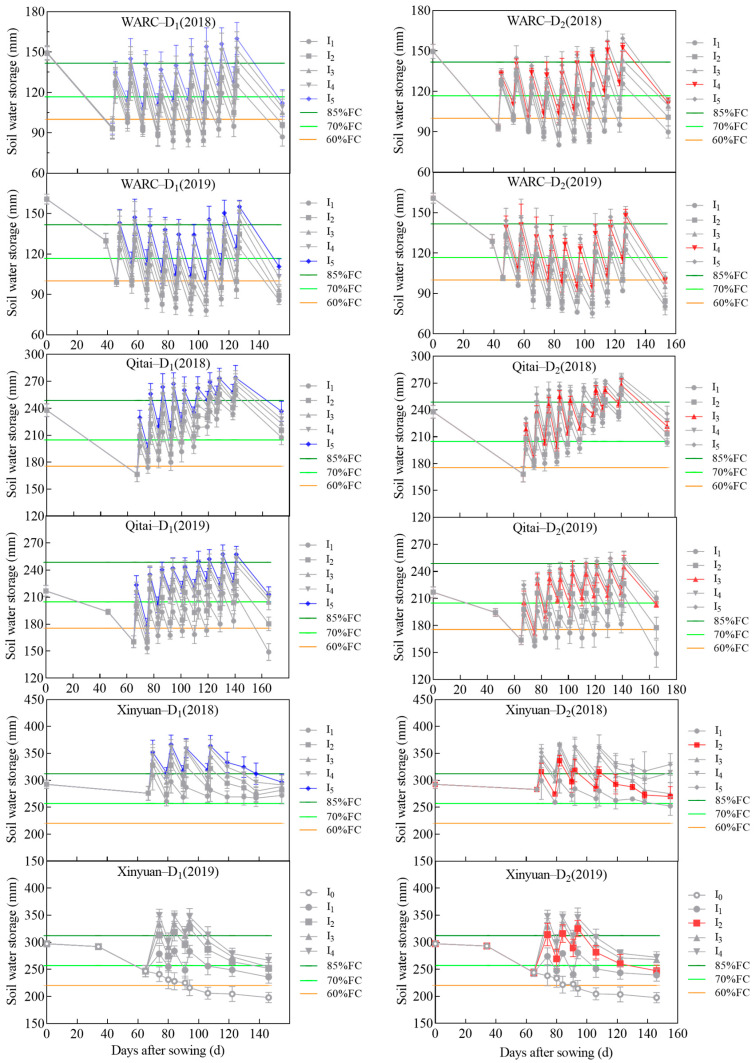
Soil water storage of maize field under different treatments at WARC, Qitai, and Xinyuan. Blue curves represent the values of the farmers’ planting patterns, and red curves represent the values of the optimization patterns.

Soil water storage (SWS) in the 0–100 cm profile exhibited distinct patterns under the two production models (Figure 9). Under the conventional farmer model (D_1_ + I_5_), SWS remained significantly above the target threshold throughout the growing season at all sites, with seasonal average SWS > 77% of field capacity (FC) at WARC, >79% FC at Qitai, and >88% FC at Xinyuan (Figure 9). In contrast, the optimized model (D_2_ + I_opt_) consistently maintained SWS close to the target of ~70% FC, with seasonal averages of approximately 73% FC at WARC, 76% FC at Qitai, and 80% FC at Xinyuan (Figure 9). The SWS under D_2_ + I_opt_ was significantly lower than under D_1_ + I_5_ at all sites (*p* < 0.05), yet never dropped below 60% FC, the inferred lower boundary for maintaining yield stability (based on dry matter and yield responses).

**Figure 10:**
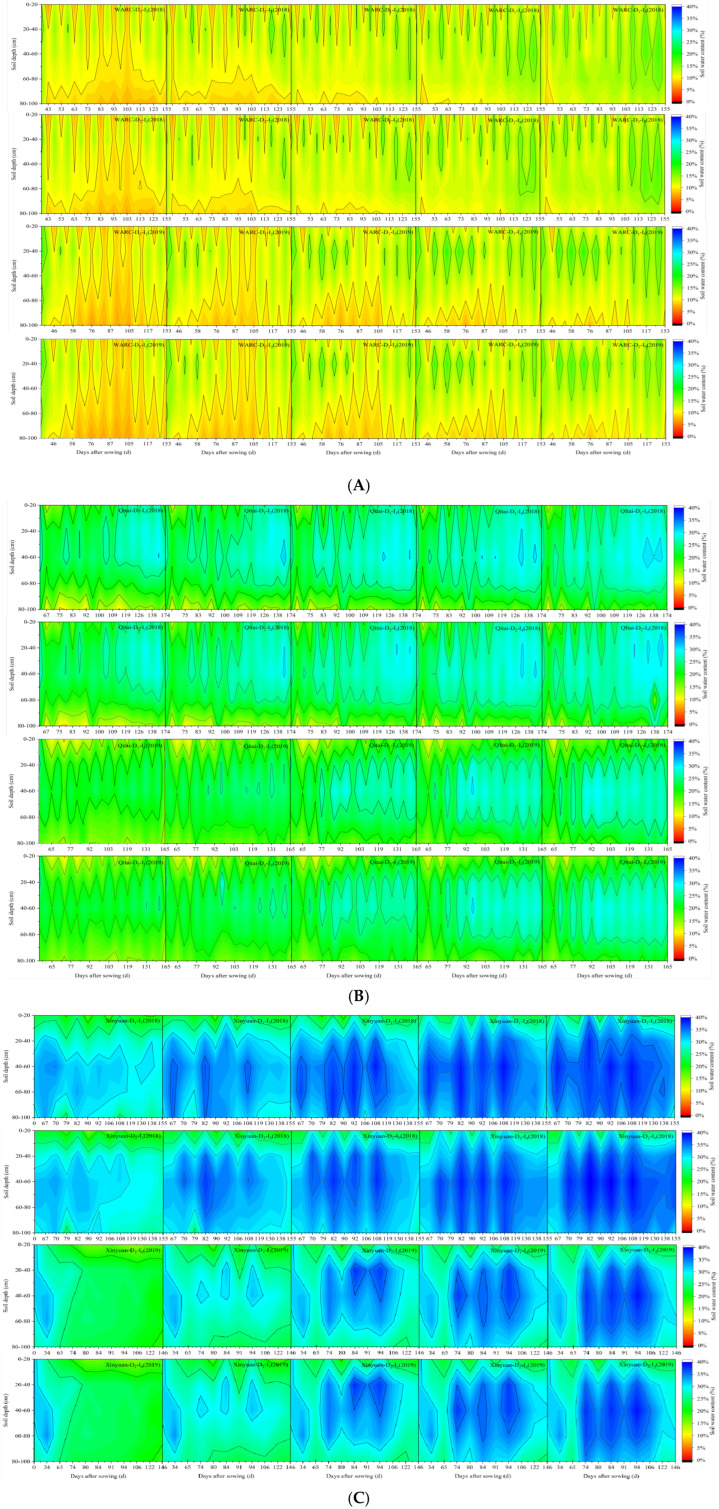
(**A**) Soil water content at the 0–100 cm soil layer under different treatments at WARC. (**B**) Soil water content at the 0–100 cm soil layer under different treatments at Qitai. (**C**) Soil water content at the 0–100 cm soil layer under different treatments at Xinyuan.

The spatiotemporal dynamics of soil water content (Figure 10A–C) further corroborate this: under the optimized model, water was effectively retained within the active root zone (0–80 cm), with minimal deep percolation below 100 cm. Collectively, these results demonstrate that the synergy of dense planting and regulated deficit irrigation operates by maintaining root zone soil moisture within an optimal range (~70% FC) (Figure 10A–C), which minimizes non-productive water losses while sustaining crop water demand.

### 2.7. Structural Equation Modeling (SEM)

**Figure 11:**
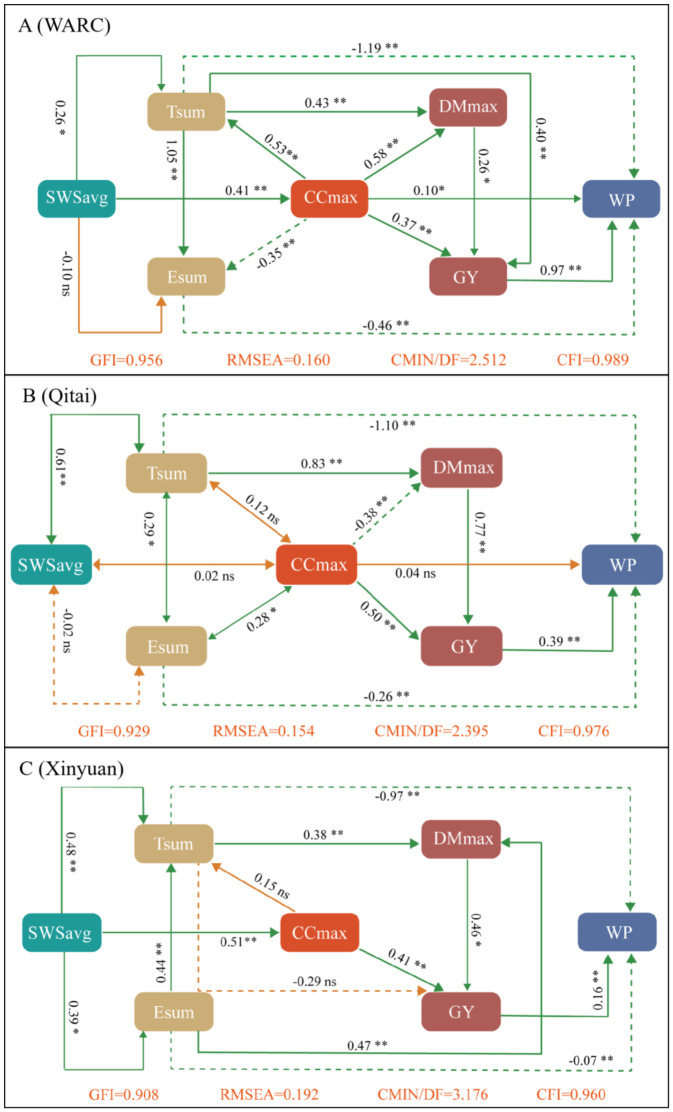
(**A**) The structural equation model of the effects of experimental treatments on WP and GY at WARC. (**B**) The structural equation model of the effects of experimental treatments on WP and GY at Qitai. (**C**) The structural equation model of the effects of experimental treatments on WP and GY at Xinyuan. WP is water productivity, GY is grain yield, SWS_avg_ is average soil water storage, Tsum and E_sum_ are total transpiration and soil evaporation during maize growing season, CC_max_ is maximum canopy coverage at silking stage, and DM_max_ is maximum dry matter accumulation at maturity stage. The solid lines and dashed lines represent the paths with significant positive and negative effects, respectively; the green and orange lines represent the paths with significant and non-significant effects, respectively. * means *p* < 0.05; ** means at *p* < 0.01; ns, means not significant.

To reveal the causal mechanisms through which planting density and irrigation amount synergistically enhance grain yield (GY) and water productivity (WP), a structural equation model (SEM) was constructed. The model analyzed the pathway relationships among key indicators, including average soil water storage (SWS_avg_), total transpiration (T_sum_), total soil evaporation (E_sum_), maximum canopy coverage (CC_max_), and maximum dry matter accumulation (DM_max_) (Figure 11). The model demonstrated good fit across all climatic zones (WARC: CFI = 0.989; Qitai: CFI = 0.976; Xinyuan: CFI = 0.960), effectively revealing significant differences in the dominant synergistic pathways among regions.

In the arid region (WARC, Figure 11A), maintaining suitable soil water directly promoted canopy development (SWS_avg_ → CC_max_ = 0.41 **). Sufficient canopy coverage became the core driver of yield by sustaining productive transpiration (T_sum_ → GY = 0.40 **) and dry matter accumulation (DM_max_ → GY = 0.26 **). Concurrently, effective canopy coverage significantly suppressed soil evaporation, directly enhancing WP (E_sum_ → WP = −0.46 **). The synergistic pathway is characterized as “water-promoted canopy → canopy-sustained transpiration/matter → evaporation suppression for efficiency gain.”

In the semi-arid region (Qitai, Figure 11B), soil water exerted a strong direct control on transpiration (SWS_avg_ → T_sum_ = 0.61 **), which in turn strongly drove dry matter accumulation (T_sum_ → DM_max_ = 0.83 **). Yield directly depended on sufficient dry matter (DM_max_ → GY = 0.77 **). Canopy coverage indirectly supported the system mainly by promoting yield (CC_max_→GY = 0.50 **) and influencing evaporation (CC_max_ → E_sum_ = 0.28 *). The improvement in WP was primarily derived from the maximal suppression of soil evaporation (E_sum_ → WP = −0.26 **). The pathway emphasizes “water-controlled transpiration → transpiration-promoted matter → simultaneous focus on high yield and evaporation suppression.”

In the semi-humid region (Xinyuan, Figure 11C), the regulatory pattern in this region was distinctly different. Soil water had a significant positive effect on canopy coverage (SWS_avg_ → CC_max_ = 0.51 **), and canopy coverage directly drove yield (CC_max_ → GY = 0.41 **). Yield itself became the strongest direct driver for enhancing WP (GY → WP = 0.39 **). Simultaneously, suppressing transpiration was another key direct pathway for improving WP (T_sum_ → WP = −0.97 **). This indicates that the core objective of optimized management in this region is not unlimited canopy expansion but rather “water-controlled canopy optimization → yield-driven efficiency → transpiration suppression for efficiency gain,” aiming to avoid excessive vegetative growth, efficiently convert water into yield, and control transpiration losses.

## 3. Discussion

### 3.1. Synergistic Effects of Dense Planting and Deficit Irrigation on Yield and WP

This study advances the high-density production system enabled by precision stage-specific regulation (HD-PSR) by identifying a quantifiable irrigation module that synchronizes yield and water productivity (WP) gains. Moving beyond isolated assessments of density or water management, we demonstrate that the synergy between high planting density and regulated deficit irrigation emerges from maintaining soil water storage (SWS) at ~70% of field capacity (FC). This threshold functions as a physiological set point that stabilizes canopy development, shifts water use toward productive transpiration (T), and sustains carbon allocation—key processes for overcoming the yield–WP trade-off in water-limited systems.

The conventional practice of low-density planting combined with excessive irrigation often results in “luxury” water consumption, where increased evapotranspiration (ET) fails to translate proportionally into higher yield, thereby depressing WP [8,9]. Our results demonstrate that the optimized paradigm fundamentally alters this relationship. The synergy stemmed from a strategic reallocation of water through non-productive soil evaporation (E) to productive plant transpiration (T). While increasing planting density inherently raises the crop’s water demand to achieve maximum yield—quantified here as an additional 5.7–35.4 mm of irrigation required per 10,000 plants·ha^−1^ increase (Figure 2)—the implementation of regulated deficit irrigation precisely met this increased demand without excess. Crucially, the higher planting density accelerated early ground cover, which in turn suppressed soil E by shading and altering the microclimate [6]. This created a positive feedback loop, where denser canopies under optimized irrigation conserved soil moisture more effectively, further improving the T/ET ratio [20,21]. This ratio is recognized as a physiological cornerstone of enhanced WP, as productive transpiration is directly linked to carbon assimilation [22,23]. The significant interaction between irrigation and density for WP at all sites statistically confirms that the combined effect is greater than the sum of their individual parts.

### 3.2. The Central Role of the ~70% FC Threshold in Coordinating Crop Physiology

The consistent association of optimal outcomes with maintaining root zone SWS at approximately 70% FC across diverse climates points to its role as a key physiological set point (Figure 9). This threshold appears to optimally balance the plant’s need for water to maintain cellular turgor (for stomatal conductance and photosynthesis) against the risks of both water logging-induced hypoxia and severe water deficit [24,25].

In arid/semi-arid areas (WARC, Qitai): The ~70% FC threshold primarily served a protective and stabilizing function. It provided sufficient water to maintain canopy development (CC_max_, Figure 6) and sustain the critical rapid phase of dry matter accumulation (V_max_, T; Figure 5) under high atmospheric demand. This is evidenced by the strong positive pathways in the structural equation model (SEM) (Figure 11A,B). Preventing SWS from dropping below ~60% FC was crucial, as crossing this lower boundary triggered significant declines in canopy cover, biomass, and yield, marking it as a carbon crisis threshold where assimilate production becomes severely impaired [26].

In the semi-humid area (Xinyuan): The dynamic was distinct. Here, the ~70% FC threshold acted primarily as a growth-regulating signal. Soil water levels approaching FC likely promoted excessive vegetative growth, potentially leading to canopy closure, reduced light penetration, and diminished radiation use efficiency at the individual plant level [27]. This explains a potential point of confusion: why did dry matter and canopy coverage increase while transpiration was reduced under the optimized model? The answer lies in the fact that, at Xinyuan, conventional over-irrigation (I_5_) drove unnecessarily high transpiration rates that did not translate into proportional yield gains. By reducing irrigation to the I_2_ level (maintaining SWS at ~70% FC), the optimized model curbed this “wasteful” transpiration without harming canopy development or dry matter accumulation—indeed, it even improved these due to better canopy structure and reduced inter-plant shading. Thus, regulating irrigation to maintain the ~70% FC threshold optimized canopy structure, prevented resource waste on excess vegetative tissue, and directed more assimilates toward grain filling, explaining the exceptionally high WP observed (Figure 11C).

This framework leverages conserved plant physiological responses to mild water deficit. Such conditions can promote root system plasticity, enhancing exploration of soil water [28], and can stimulate osmotic adjustment and the remobilization of preanthesis assimilates (e.g., stem starch) to support grain filling under moderate post-silking water limitation [29,30]. Our optimized irrigation regime appears to have gently triggered these adaptive mechanisms—root plasticity and carbon partitioning—without imposing damaging stress, thereby “training” the crop for more efficient water use.

### 3.3. Comprehensive Analysis of Causal Paths of GY and WP Synergetic Promotion Based on SEM

Structural equation modeling (SEM) provided robust, quantitative evidence for the associative pathways linking soil water, canopy traits, and yield/WP. While SEM cannot prove causation, it reveals mechanistically plausible pathways consistent with physiological knowledge [31]. The analysis showed systematically divergent pathways underpinning the synergy across the aridity gradient (Figure 11). We emphasize that all path coefficients represent associations, not definitive causal relationships.

At the arid site (WARC): The dominant pathway (SWS → CC → T → GY) suggests that under high atmospheric demand, maintaining a robust canopy is paramount for sustaining productive transpiration—the physiological link to yield formation [32,33]. This aligns with the concept that maximizing the T/ET ratio is a cornerstone of high WP in water-limited environments [34].

At the semi-arid site (Qitai): A strong transpiration mediated carbon capture pathway (SWS → T → DM → GY) was observed. This underscores the critical role of sustained transpirational flow in driving assimilate accumulation, particularly when vapor pressure deficit is a significant environmental modulator [35,36]. The extreme sensitivity of WP to the suppression of soil evaporation further emphasizes that, in semi-arid zones, minimizing non-productive water losses is often the primary route to efficiency gains [37].

At the semi-humid site (Xinyuan): A yield-driven efficiency pathway (CC → GY → WP) prevailed. Here, the primary challenge shifts from water scarcity to avoiding the diminishing returns of excessive water availability, which can lead to canopy closure, reduced light penetration, and lower harvest indices [38,39]. In such contexts, high yield itself becomes the most effective lever for improving WP, as fixed metabolic costs are amortized over a larger sink.

Therefore, the SEM analysis provides mechanistically plausible validation for the irrigation core of the HD-PSR framework. It demonstrates that precision water regulation, anchored in a generalizable soil moisture threshold (~70% FC), is foundational to unlocking the stable synergy between intensification (density) and sustainability (efficient water use). This offers a scalable, physiologically informed model for sustainable intensification in water-limited agroecosystems [1].

### 3.4. Implications, Limitations, and Future Perspectives

This study provides a robust, physiologically grounded irrigation guideline for high-density maize systems under mulch drip irrigation in arid to semi-humid zones. However, several limitations should be acknowledged.

The experiments were conducted in only one region (Xinjiang, China) with a single maize hybrid (Xianyu 335) under plastic mulch and drip irrigation, so the generalizability of the proposed ~70% FC threshold to other genotypes, soil types, irrigation methods, or unmulched conditions requires further testing. The analysis is based on only two growing seasons (2018–2019), which limits our ability to capture interannual climate variability.

To isolate the pure effects of planting density and irrigation regime on yield and water productivity (WP), we applied nitrogen without restriction—specifically, a total of 600 kg N ha^−1^ (basal + topdressing). This high rate was deliberately chosen to completely eliminate nitrogen limitation for the high-density (12 × 10^4^ plants ha^−1^) target yield of up to 18 Mg ha^−1^. However, we fully recognize that this rate exceeds the crop’s physiological optimum and may have induced redundant vegetative growth and luxury transpiration, potentially leading to an overestimation of WP gains attributed to the optimized irrigation regime. Consequently, the results may not directly apply to nutrient-limited systems, and the proposed ~70% FC threshold should be validated under optimized nitrogen–water co-management before field-scale recommendation.

Additionally, we did not perform an economic analysis (e.g., seed costs, infrastructure investment), which is necessary to assess the practical adoptability of the proposed strategy. Despite these constraints, the observed consistency of the synergistic yield–WP response across three contrasting climates provides robust evidence for the proposed soil water threshold as a physiologically based guideline, pending further validation under broader conditions.

Future research should focus on two fronts to address generalizability and mechanistic depth. First, mechanistic crop modeling (e.g., APSIM or AquaCrop) calibrated with this dataset could extrapolate these findings, test the threshold under projected climate variability, and optimize it for other cultivars [40,41,42]. Second, targeted physiological investigations could further elucidate the specific signals (e.g., abscisic acid gradients, root to shoot signaling) triggered by the ~70% FC moisture regime that coordinate the observed improvements in canopy development and carbon partitioning. In summary, this study establishes that the synergy between dense planting and deficit irrigation is governed by a precise soil water threshold. By positioning this finding as the water regulation core of the HD-PSR framework, we provide a scalable, physiologically grounded strategy to simultaneously raise maize productivity and water use efficiency in water-limited agroecosystems.

## 4. Materials and Methods

### 4.1. Site Description

**Figure 12:**
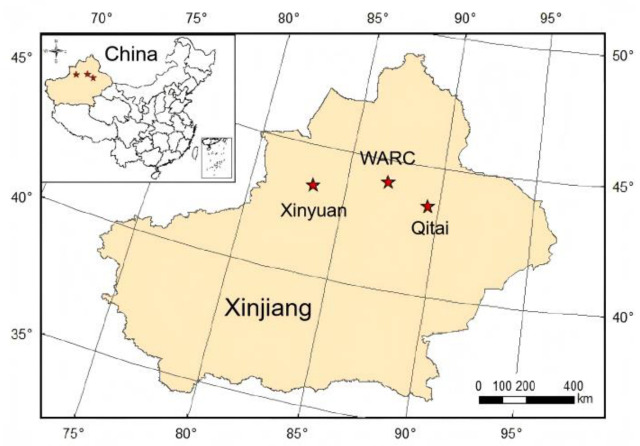
Map of the WARC, Qitai, and Xinyuan in Xinjiang, Northwestern China.

Field experiments were conducted during the 2018 and 2019 maize growing seasons at three sites in Xinjiang, China: the Western Agricultural Research Center of the Chinese Academy of Agricultural Sciences (WARC, 87°11′59″ E, 44°09′33″ N; 470 m a.s.l.), Qitai (89°28′42″ E, 43°29′15″ N; 1021 m a.s.l.), and Xinyuan (83°19′50″ E, 43°27′37″ N; 817 m a.s.l.) (Figure 12).

We adopted the widely used Chinese agro-climatic classification system based on the relationship between annual precipitation (P) and potential evaporation (ET_0_), together with absolute precipitation thresholds. According to this system: arid regions—P < 200 mm, and P < ET_0_, requiring full dependence on irrigation for crop production; semi-arid regions—200 mm ≤ P < 400 mm, and P < ET_0_, though supplementary irrigation is necessary; and semi-humid regions—400 mm ≤ P < 800 mm, and P < ET_0_, where rainfed agriculture is possible but irrigation stabilizes yield.

By applying this classification to our sites, the WARC had a mean annual precipitation of 141 mm, mean annual ET_0_ of ~2650 mm, and P < 200 mm; thus, it was classified as arid. Qitai Farm received 269 mm of annual precipitation with annual ET_0_ of ~1386 mm, satisfying 200 mm ≤ P < 400 mm and P < ET_0_, and was therefore semi-arid. Xinyuan, located in the Ili River Valley, had a mean annual precipitation of 476 mm (P > 400 mm) despite its annual ET_0_ (1286 mm) exceeding precipitation; accordingly, it was classified as semi-humid in this study.

### 4.2. Meteorological and Soil Data

The physicochemical properties of the 0–100 cm soil layer at each site are provided in Table 2.

The meteorological data of the maize growing season from 2018 to 2019 are shown in Table 3 in three ecological regions.

### 4.3. Experimental Design

**Figure 13:**
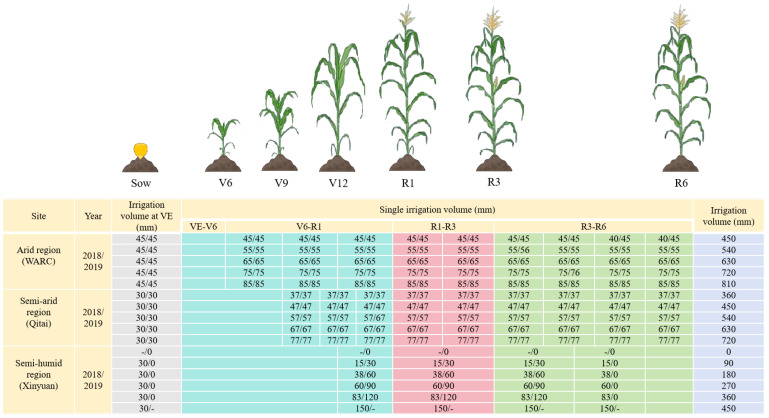
Irrigation treatment, irrigation time, and single irrigation amount at WARC, Qitai, and Xinyuan.

The maize variety Xianyu 335 was used across all experimental sites. A split-plot design was adopted, with irrigation amount assigned to the main plots and planting density to the subplots. Planting densities included local farmers’ practice density (D_1_, 9 × 10^4^ plants ha^−1^) and high density (D_2_, 12 × 10^4^ plants ha^−1^). The crop was sown in alternating wide–narrow rows (70 cm and 40 cm) [13]. Irrigation was supplied using a surface drip irrigation system, where the drip lines were placed on the soil surface between rows and subsequently covered with plastic film. The maximum irrigation treatment (I_5_) corresponded to local farmers’ conventional practice (full irrigation). Four reduced irrigation treatments (I_1_–I_4_) were established by progressively decreasing I_5_ by 90 mm increments: at WARC (arid)—I_1_ = 450, I_2_ = 540, I_3_ = 630, I_4_ = 720, and I_5_ = 810 mm; at Qitai (semi-arid)—I_1_ = 360, I_2_ = 450, I_3_ = 540, I_4_ = 630, and I_5_ = 720 mm; and at Xinyuan (sub-humid)—I_1_ = 180, I_2_ = 270, I_3_ = 360, I_4_ = 450, and I_5_ = 540 mm. The 90 mm step was chosen based on preliminary trials indicating that this increment produced distinct water stress levels without causing severe yield collapse, and it aligned with typical single irrigation amounts used by local farmers [12]. Irrigation treatments were consistent across 2018 and 2019 at WARC and Qitai. In Xinyuan, compared with the 2018 preliminary season, the 0 mm treatment (I_0_) was introduced, while the 540 mm treatment was removed in 2019. All treatments were replicated three times. The exact dates and single event amounts are shown in Figure 13.

### 4.4. Field Management

Each experimental plot measured 165 m^2^ (15 m length × 11 m width). To prevent lateral water movement, a 1 m deep waterproof membrane was buried around each plot, and a 1 m wide buffer area was established. Independent water meters installed for each plot ensured precise measurement and control of irrigation water application.

Maize was irrigated immediately after sowing (within 48 h) to ensure uniform and rapid germination, with specific irrigation amounts detailed. The first irrigation event during the growing season was applied at the jointing stage (V6) in WARC, the 10-leaf stage (V10) in Qitai, and the 12-leaf stage (V12) in Xinyuan. Subsequent irrigation intervals were 8–9 days in WARC and Qitai, and 12–15 days in Xinyuan (Figure 13).

Fertilizer management: Based on initial soil N, P, and K contents in the 0–30 cm profile, pre-sowing base fertilizers were applied—urea (150 kg N ha^−1^), diammonium phosphate (225 kg ha^−1^), and potassium sulfate (75 kg K_2_O ha^−1^). To achieve a target maize yield > 15 Mg ha^−1^ (and up to 18 Mg ha^−1^ under high density) and to completely eliminate nitrogen limitation, additional urea was applied as topdressing, totaling 600 kg N ha^−1^ across all treatments. The topdressing was split into three applications at the six-leaf (V6), twelve-leaf (V12), and tasseling (VT) stages in a ratio of 3:4:3, following local high-yield practice. This N rate exceeds the crop’s physiological optimum. However, it was deliberately chosen to isolate the pure effects of planting density and irrigation regime on yield and water productivity. Weeds, diseases, and pests were controlled throughout the growing season following local government recommendations.

### 4.5. Measurement Indices

#### 4.5.1. Soil Water Content (SWC) and Storage (SWS)

Soil water content (SWC) was determined gravimetrically from samples collected prior to sowing. Throughout the growing season, from sowing to harvest, SWC was monitored at 7–10-day intervals using Time Domain Reflectometry (TDR) (TRIME-PICO-BT, Cologne, Germany). During each monitoring event, the TDR tubular probe was inserted into pre-installed access tubes to obtain measurements at specified depth intervals (0–20, 20–40, 40–60, 60–80, and 80–100 cm). At each depth within a tube, three replicate readings were taken and averaged to represent the volumetric water content for that specific depth and time point.

The soil water storage (SWS, mm) represents the sum of the water storage in the entire soil profile (0–100 cm). It was calculated using the equation provided in Equation (1).

(1) $$\mathrm{SWS}=\left(\sum_{i}^{n}\mathrm{SWCi}/100\right)\times 20\;$$

where SWCi is the volumetric water content of the soil (%), while i indicates soil depths of 0–20, 20–40, 40–60, 60–80, and 80–100 cm.

Field capacity (FC) was determined in situ using the ponding method. SWSFC refers to the soil water storage when the soil water content reaches the FC.

#### 4.5.2. Evapotranspiration (ET), Evaporation (E), and Transpiration (T)

ET consists of two parts: soil evaporation (E) and plant transpiration (T).

The actual crop evapotranspiration (ET, mm) during the growing season was calculated using Equation (2) [8].

(2) $$\mathrm{ET}=P+I+C_{r}-D_{p}-R_{f}\pm \Delta S\;$$

where ET is actual crop evapotranspiration (mm); P is seasonal precipitation (mm); I is total irrigation amount (mm); C_r_ is the capillary rise (mm); D_p_ is deep percolation (mm); R_f_ is runoff (mm); and ΔS is the change in soil water storage before sowing and after harvest (mm). In Equation (1), C_r_ was set to zero due to a deep groundwater table (>10 m). R_f_ was considered negligible given the level topography of the experimental plots. Additionally, D_p_ was taken as zero because the soil water content beneath the 80–100 cm layer never reached FC on any sampling date across the WARC, Qitai, and Xinyuan sites.

Soil evaporation (E) was measured with a mini-lysimeter [43]. The mini-lysimeter consisted of two parts: an outer cylinder with steel pipe (inner diameter, length, and thickness were 10 cm, 15 cm, and 1 mm, respectively) and an inner cylinder with PVC (inner diameter, length, and thickness were 12 cm, 15 cm, and 1 mm, respectively). The difference in the weight of the mini-lysimeter over two consecutive days was the amount of soil evaporation [12]. For each specific measurement, the inner cylinder was first pushed vertically into the soil and drawn out to obtain the undisturbed soil. Then, the bottom of the inner cylinder was sealed with aluminum foil and the soil was weighed. And finally, the inner cylinder was put into the outer cylinder and fixed between the rows of corn with a wide interval. An electronic scale (DS6000, Shouheng, Beijing, China) with a precision of 0.01 g was used to measure the mass. Three mini-lysimeters were placed in each plot and weighing was performed every day at 17:00. The original soil in the mini-lysimeters was replaced every two days in order to maintain the same soil moisture condition for each plot. After a precipitation or irrigation event, the soil was replaced. E was calculated using Equation (3).

(3) E=α×Δm 

where E is soil evaporation (mm); α is a conversion factor (0.127 mm); and Δm (g) is the mass difference between mini-lysimeters in one unit of time.

T was calculated using Equation (4).

(4) T=ET−E 

#### 4.5.3. Canopy Coverage (CC)

After maize seedling emergence, the length (L) and maximum width (W) of all leaves were measured on five randomly selected plants at the following growth stages: V6, V12, R1, R3, 40 days after silking (R1 + 40), 50 days after silking (R1 + 50), and physiological maturity (R6). The leaf area index (LAI) was calculated using Equation (5) for expanded leaves and Equation (6) for non-expanded leaves [44].

(5) $$\mathrm{LAI}=C\times M\times \sum \left(L\times W\right)/A\;$$

where C refers to the coefficient (unfolded leaves are 0.75, and folded leaves are 0.5), while M and A are the number of plants in each plot and the area of the plot (m^2^), respectively.

The calculation equation for CC was calculated using Equation (6) [45].

(6) $$\mathrm{CC}=1.005\times {\left[1-\exp\left(-0.6\mathrm{LAI}\right)\right]}^{1.2}\;$$

#### 4.5.4. Dry Matter

The dry matter of five plants was measured at the SOW, V6, V12, R1, R3, R1 + 40, and R6 stages. The accumulation of dry matter during the growth period of maize was fitted using the logistic function in Equation (7).

(7) $$\mathrm{DM}={\mathrm{DM}}_{\max}/\left(1+ae^{-\mathrm{bt}}\right)\;$$

where DM is the dry matter and t is the number of days after SOW, V6, V12, R1, R3, R1 + 40, and R6.

The first and second derivatives of the logistic equation were computed to determine growth dynamics parameters. These included: the maximum dry matter (DM_max_) accumulation rate (V_max_), the start (t_1_) and end (t_2_) of times of peak accumulation, and the duration (T) of the rapid dry matter accumulation phase [46].

#### 4.5.5. Grain Yield

At physiological maturity, a 66 m^2^ area from the middle six rows of each plot was manually harvested. The total numbers of plants and ears were recorded.

#### 4.5.6. Water Productivity

Water productivity (WP, kg·ha^−1^·mm^−1^) based on ET and grain yield was calculated according to the following equation [47].

(8) $$\mathrm{WP}=\mathrm{GY}/ET_{c}\times 100\;$$

### 4.6. Statistical Analysis

ANOVA (SPSS 26.0) with Duncan’s multiple range test (α = 0.05) was used. Quadratic regression between GY and irrigation amount was performed. Structural equation modeling (SEM) was conducted using the “lavaan” package in R (version 4.4.2). SEM path coefficients were estimated with maximum likelihood; model fit was evaluated by χ^2^, CFI, and RMSEA. Figures were generated using GraphPad Prism (v9.5), Origin 2021, and R 4.4.2.

## 5. Conclusions

Shifting from conventional farmers’ practice (low density + excessive irrigation) to an optimized strategy (high density + regulated deficit irrigation) synergistically enhanced both maize grain yield (>15.0 Mg·ha^−1^) and water productivity (by 6.8–61.5% across climates). This synergy, which breaks the common yield and WP trade-off, was primarily governed by maintaining root zone soil water storage at approximately 70% of field capacity. This quantifiable threshold ensured sufficient canopy coverage and dry matter accumulation for yield stability while simultaneously promoting a favorable shift in water partitioning—reducing non-productive soil evaporation in arid/semi-arid regions and curbing excessive transpiration in the sub-humid area. Structural equation modeling revealed associative pathways that differed by climate, but these all converged on the 70% FC set point. Thus, strategic crop densification guided by the ~70% FC soil water threshold provides a robust, transregionally validated framework for sustainable intensification in water-limited maize production systems.

## Figures and Tables

**Table 1:** Interaction influence of year, planting density, and irrigation on evapotranspiration (ET), transpiration (T), and soil evaporation (E) at WARC, Qitai, and Xinyuan. ** *p* < 0.01; ns, no significance.

Site		ET	T	E
	Source of Variation			
WARC	Year	**	**	**
	Planting density	ns	**	**
	Irrigation	**	**	**
	Year × Planting Density	ns	**	**
	Year × Irrigation	ns	**	**
	Planting Density × Irrigation	ns	ns	ns
	Year × Planting Density × Irrigation	ns	ns	ns
Qitai	Year	**	**	**
	Planting Density	ns	**	**
	Irrigation	**	**	**
	Year × Planting Density	ns	ns	ns
	Year × Irrigation	**	**	ns
	Planting Density × Irrigation	ns	ns	ns
	Year × Planting Density × Irrigation	ns	ns	ns
Xinyuan	Year	ns	**	ns
	Planting Density	ns	ns	**
	Irrigation	**	**	**
	Year × Planting Density	ns	**	ns
	Year × Irrigation	ns	**	ns
	Planting Density × Irrigation	**	**	ns
	Year × Planting Density × Irrigation	ns	**	ns

**Table 2:** Mean soil physicochemical properties within the 0–100 cm profile across experimental sites in 2018 and 2019. Undisturbed soil samples were collected from depth intervals of 0–20, 20–40, 40–60, 60–80, and 80–100 cm. Soil pH was measured in a 1:5 (soil:deionized water) suspension using a PHS-3C pH meter (Shanghai Kangyi Inc., Shanghai, China). Electrical conductivity (ECe) was determined in a 1:5 (soil:deionized water) extract using a conductivity meter (FE30-K, Mettler-Toledo, Shanghai, China). Field capacity is expressed as gravimetric water content (%, kg water per kg dry soil). Bulk density was determined using the core method; samples were oven-dried at 105 °C to constant weight, and bulk density (g cm^−3^) was calculated as the dry soil mass divided by the cutting-ring volume. All measurements were performed with three replicates per treatment.

Station	Soil Properties					
	Silt + Clay (<0.01 mm) (%)	pH	ECe (uS m^−1^)	Organic Matter Content (g kg^−1^)	Field Capacity (%)	Bulk Density (g cm^−3^)
WARC	29.9	7.7	1577.9	4.0	14.1	1.5
Qitai	39.1	8.4	115.4	6.6	22.1	1.3
Xinyuan	33.3	8.1	261.7	17.3	23.3	1.4

**Table 3:** Meteorological data of maize growth period for the experimental stations in 2018 and 2019.

Station	Year	Meteorological Conditions			
		Cumulative Sunshine Hours (h)	≥10 °C Accumulated Temperature (°C)	Precipitation (mm)	Average ET_0_ (mm d^−1^)
WARC	2018	2251.0	3379.1	67.40	5.4
	2019	2150.5	3517.1	122.30	5.7
Qitai	2018	2515.5	2880.0	209.10	3.6
	2019	2391.0	2935.5	138.30	3.6
Xinyuan	2018	2259.5	2949.0	283.10	5.2
	2019	2184.0	3021.6	252.20	5.4

## Data Availability

The original contributions presented in the study are included in the article. Further inquiries can be directed to the corresponding authors.
